# Disentangling rodent behaviors to improve automated behavior recognition

**DOI:** 10.3389/fnins.2023.1198209

**Published:** 2023-07-11

**Authors:** Elsbeth A. Van Dam, Lucas P. J. J. Noldus, Marcel A. J. Van Gerven

**Affiliations:** ^1^Department of Artificial Intelligence, Donders Institute for Brain, Cognition and Behaviour, Radboud University, Nijmegen, Netherlands; ^2^Noldus Information Technology BV, Wageningen, Netherlands; ^3^Department of Biophysics, Donders Institute for Brain, Cognition and Behaviour, Radboud University, Nijmegen, Netherlands

**Keywords:** action recognition, deep learning, continuous video analysis, behavior recognition, rodent behavior

## Abstract

Automated observation and analysis of behavior is important to facilitate progress in many fields of science. Recent developments in deep learning have enabled progress in object detection and tracking, but rodent behavior recognition struggles to exceed 75–80% accuracy for ethologically relevant behaviors. We investigate the main reasons why and distinguish three aspects of behavior dynamics that are difficult to automate. We isolate these aspects in an artificial dataset and reproduce effects with the state-of-the-art behavior recognition models. Having an endless amount of labeled training data with minimal input noise and representative dynamics will enable research to optimize behavior recognition architectures and get closer to human-like recognition performance for behaviors with challenging dynamics.

## 1. Introduction

Automated observation and analysis of behavior is important to facilitate progress in many fields of science, especially in behavioral studies for neurological disorders or drug discovery, where rodents (mice and rats) are still the most commonly used model animals in preclinical research. With increasingly large image datasets and computational hardware capacity, we have seen a tremendous progress in pose estimation for many different animal species (Mathis et al., 2018; Lauer et al., 2022). In behavior recognition, the progress has not been that evident. Available systems recognize behaviors with a reliability of around 70–75% (Dam et al., 2020), or are trained and tested on footage from the same recording session, for a limited set of specific behaviors. However, in order to be useful in behavioral research, automated systems that can recognize behavioral activities must be able to recognize them independent of animal genetic background, drug treatment or laboratory setup. To match human-level performance in annotating behavior, we need to improve accuracy, robustness and genericity of automated systems. Accuracy means good precision and recall per behavior, robustness means consistent accuracy across experimental setups, and genericity means that the same method is applied to all behaviors. Three approaches are at hand. First is to standardize laboratory setups, i.e., the test environment in which the animals are observed (Grieco et al., 2021). This limits the variance but leaves the animal- and treatment-related variation. Second is to aim for quick adaptation of the recognition system toward a new setup with minimal annotation effort, i.e., fine-tuning or retraining. This requires new ground truth data and brings back the manual annotation task for a significant number of video segments. Moreover, and more importantly, researchers who need to compare animal behavior between treatment groups need one measurement system instead of separately trained observation models. The third approach is to explicitly strive for generic recognition with robust methods, which is in principle possible as humans can do so.

In this paper, we investigate where we stand with respect to the goal of generic recognition, and what is needed when we raise the bar for future automated behavior recognition, that is, (1) to recognize ethologically relevant behaviors, (2) recognize behaviors robustly across experimental setups, and (3) recognize new behaviors with limited data and fine-tuning effort.

Robustness across experimental setups requires that the system can handle variation in three aspects, namely appearance, behavior execution, and behavioral sequence. For the behavior class performed, the appearance of the animal is irrelevant, i.e., whether the animal is white or black, thick or slim, long or short-haired. The same applies to the appearance of the environment, such as the walls, floor, feeder, drink spout or enrichment objects. While their presence may enable or limit certain behaviors, their color and texture should not affect recognition. Behavior recognition should also be immutable to how behaviors are executed, i.e., differences in event duration, pace and subbehavioral pattern. In addition to the usual event variations, behavior execution varies by physical or emotional state, and by individual animal, depending on strain, gender, age, history and medication. Furthermore, execution varies due to different layout of the environment, such as the size of the cage or the height of the drink spout. The third aspect for which automated recognition systems need to be robust is the sequence of the behaviors performed, as the treatment of animals affects the frequencies of specific behaviors. Behavior recognition systems that use history or recurrence such as hidden Markov models (HMMs), recurrent neural networks (RNNs) or 3D convolutional neural networks (3D-CNNs) train on temporal context and hence on behavioral context, and will have difficulty to recognize the behavior events when applied in a different context.

There are multiple ways to increase robustness of behavior classification systems. The best way is to train on larger and more diverse datasets. This is costly and it is not always possible to cover all experimental diversity beforehand. Alternatively, we can factor out variance up front by normalizing the input. By using tracked body points we can focus on the poses and dynamics, and solve most of the appearance bias (Graving et al., 2019). Furthermore, there are training “tricks” to improve a model's internal robustness, such as dropout and variational encoding of latent variables (Goodfellow et al., 2016). We can also add variance by augmentation of the input, altering the input in ways that leave the behavior intact. Most data augmentation methods used are augmentations of appearance, such as size, scale or pixel intensity (Krizhevsky et al., 2017).

Behavior execution differences and behavior sequence differences are differences in dynamics. We believe that focus on variation in dynamics can improve behavior modeling substantially. If we can normalize and augment the behavior execution and behavior sequence, classification will be more robust. Stretching and folding the time-series to alter the speed and intensity of the movement is one way, but we can also vary the sequence of the behavior events as well as the subbehavioral pattern. To vary the sequence of the behaviors we need to detect the events and how they follow each other. To vary the subbehavioral patterns per behavior, we need to understand the type and characteristics of the possible subbehaviors and how they are combined. We give examples of composite rodent behaviors in Section 3.1.2. We further expect that breaking down composite behaviors into subbehaviors will also highlight subtle yet essential constituents and thereby will increase the detection accuracy of behaviors that are otherwise too difficult to separate from behaviors that are alike and more frequent.

The main idea of this paper is that acknowledging the hierarchical and composite structure of behavior can bring automated behavior recognition to the next level and a step closer to human-like annotation performance. If we could leave out the appearance variation and measurement errors and if we had endless amount of training data, to what extent are state-of-the-art networks able to model behavior dynamics?

We illustrate and explain three types of composite behaviors in Section 3.1. These compositions are present in the rat dataset described in Section 3.2.1. Next, we describe an artificial dataset that contains these compositions in an abstracted form and can be used to study the limits of automation models without input noise or lack of data (Section 3.2.2). Finally, we present behavior recognition results on both the rat and the artificial data in Section 4 and draw conclusions in Section 5.

## 2. Related work

### 2.1. Supervised behavior recognition

An effective recipe for training a recognition system is to record a dataset, annotate it and use supervised learning to train a classifier to recognize the behaviors. The classifier iteratively finds the best optimization path to get as close to the ground truth as it can, using all the cues it can find. Hence, the quality and robustness of the resulting classifier is always dependent on the representational value of the data trained on. In order to be robust to using cues that are only coincidentally or concurrently related to the behavior classes, data augmentation is applied to the input: typically, image transformations like flipping, scaling, and rotation. Deep learning models are very good at finding informative cues, but this also means they are sensitive to using cues that only apply within the training dataset. In almost all studies that describe behavior recognition systems, the test set is recorded in the same setup, with animals from the same strain and treatment as those in the training set. Previous work shows that although deep models can reach better performance than conventional methods, the performance is less transferable to different experiment settings (Dam et al., 2020). Supervised methods that have been applied are conventional methods as bag-of-words (Dollár et al., 2005), Bayesian classification (Dam et al., 2013) or tree-based classifiers used in MARS (Segalin et al., 2021) and SimBA (Nilsson et al., 2020). Perez and Toler-Franklin (2023) provide an overview of CNN-based approaches, such as 2D, Two Stream networks and 3D-CNNs, often combined with recurrent head to model the temporal dependencies. In recent years, major advances in deep learning classification are made using Transformer architectures that are designed to pick up the most relevant context without constraints on how far away that context is. Sun et al. (2022) report that multiple Transformer-derived networks applied to trajectory data improve the classification of social rodent behavior.

### 2.2. Data-driven approaches

During the past 10 years, data-driven approaches have been presented that learn the constituent modules of behavior from the data itself. MoSeq from Datta Lab introduced behavior syllables or motifs as behavior components (Datta, 2019) and uses autoregression filters for classification (Wiltschko et al., 2020; Costacurta et al., 2022). TREBA (Sun et al., 2021), and VAME (Luxem et al., 2022) use self-supervised learning with recurrence on sliding temporal windows to create latent representations that are used as input in supervised downstream tasks. These methods are capable of accurately predicting phenotypes and behaviors from videos withheld from the training dataset. Self-supervision is very useful when the amount of training data is small compared to the network complexity, and in discovering new significant behavior motifs or patterns. For image classification tasks, Newell (2022) showed that, with self-supervised pretraining, the top accuracy plateau is reached faster and with less data. Nonetheless, as in supervised training, accuracy increase stops around 75–80% (Sun et al., 2022). What most models have in common is the assumption that behavior consists of a sequence of behavior states and that the subject switches from one state to the next. The underlying assumption is that states can be inferred either statistically by learning the underlying state-switching process from the observed samples (HMMs), or by sliding window classification.

### 2.3. Hierarchical approaches

Other research recognizes that behavior can be looked upon at different levels and different scales, and that detection can be improved when models are trained at multiple hierarchical levels simultaneously. Gupta and Gomez-Marin (2019) show that worm behavior is organized hierarchically and derive a context-free grammar to model this. Casarrubea et al. (2018) apply T-pattern analysis to study the deep structure of behavior in different experimental contexts. Kim et al. (2019) introduce a variational approach to learn hierarchical representation of time-series on navigation tasks. Finally, Luxem et al. (2022) detect behavioral motifs in an unsupervised manner and let human experts assign labels to communities of these motifs obtained from motif traversal analysis. Recent work that most closely resembles our representation of hierarchical structure in rodent behavior is that of Weinreb et al. (2023). It builds on Moseq and extends the auto-regressive model (AR-HMM) by Switching linear dynamical systems (SLDS). They distinguish three hierarchical levels, namely behavior syllables, pose dynamics and keypoint coordinates. Their main purpose however is to denoise the input that contains erroneous keypoint jitter introduced by failing tracking.

## 3. Materials and methods

### 3.1. Behavior

In the following we provide a description of the constituents that make up behavior, give different examples of composite behavior and describe other factors that make automated behavior recognition non-trivial. We derived these constituents and compositions after visual inspection of the failures of rat behavior classification that we report in Section 4.1, as well as from the results on various other datasets reported over the years by users of the keypoint-based behavior recognition module RBR from Dam et al. (2013).

#### 3.1.1. Behavior constituents

**Figure 1:**
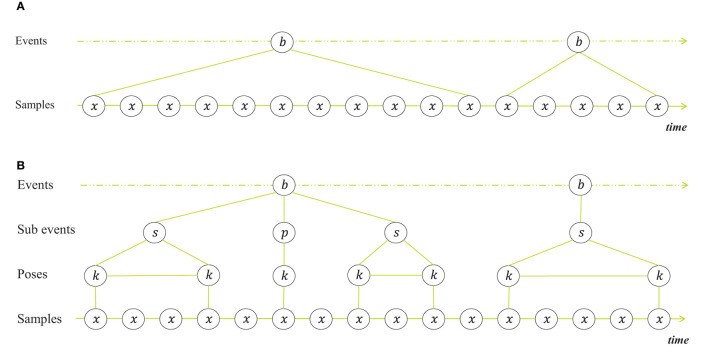
Different representations of behavior. **(A)** Behavior seen as switching states with successive behaviors *b* at event level and observations *x* at sample level (one for every time-step). **(B)** Behavior seen as hierarchically structured constituents and transitions, with two intermediate levels, namely a sub-event level and pose level. The sub-event level contains state events *s* and point events *p*. The pose level contains key poses *k*. Key poses are body postures that are held by the animal during one sub-event. The intermediate samples between successive sub events are transition samples between different key poses.

Figure 1A shows a representation of behavior seen as switching states. The samples are the observed poses, extended with derived features at the consecutive timestamps. It is implied that all observations are related to a single behavior state, and that state switches are abrupt. This is the way behavioral data is labeled that is used as ground truth for training recognition systems and that the system gets to see either one-by-one or in a sliding window with fixed length. However, when we as humans annotate behavior, we evaluate the samples differently and distinguish more than switching states. Subjective experience suggests that we predict future motion, and only take a closer look when we see deviation of what we expect, regardless of the subject or the behaviors at hand. This interpretation of the human brain as a prediction machine is supported by research in cognitive neuroscience (Keller and Mrsic-Flogel, 2018; Heilbron et al., 2022). We seem to build a belief about the goal pose and intentional state of the subject, based on the observed poses over time. When what we see no longer resembles our belief, we take a closer look, in order to revise our belief. That is, we go through the following stages of observation and inference: The subject displays behavior A - the subject no longer displays behavior A - the subject is in transition to another behavior - the subject is in transition toward either behavior B, C or D - the subject is doing behavior B. We evaluate the consecutive poses until we see that the subject arrives at a new key pose and infer the behavior from that. Sometimes we have to wait for a sequence of key poses before a decision can be made. In a transition between behaviors, the intermediate poses are merely pose changes to get from one key pose to another. They are necessary because subjects can only move around in space and time in a continuous manner. Yet, they do not define the behaviors, but are defined entirely by the previous and the next key pose. The constituents that form behavior are therefore not only states that determine the samples. Apart from states, we can also distinguish transitions, key poses with no duration and sequential combinations of these.

With this is mind we propose a new representation of behavior, shown in Figure 1B. It shows a representation of behavioral components and how they can be combined, which resembles what we see when we annotate behavior. While we are labeling the events, we perceive behavior as a sequence of state events, point events and transitions. State events are defined by key poses with a certain variation and duration, whereas point events are defined by key poses with zero or minimal duration. Note that we use point event slightly different than is common among ethologists, who use point event to indicate that the duration is not relevant for analysis. Here we want to emphasize that the behavior is characterized by a momentary key pose. Transitions are the transitional movements between different key poses (also known as movement epenthesis). Behaviors are combinations of these constituents. If we can build automated models that can detect these constituents, we can improve the recognition.

Finally a note on what should not be modeled, namely the dependencies between the behaviors at the top-level. We need to make sure that the recognition of a behavior is not dependent on the occurrence of specific previous behavior events. The behavior transition matrix is an output of an experimental test and this information should not be used during training to optimize the recognition, for if the sequence changes because of treatment effects, the detection will be hampered. In practice this means that we must have a sufficient amount of diverse training data, either by collection or augmentation.

#### 3.1.2. Three examples of composite rodent behavior

**Figure 2:**
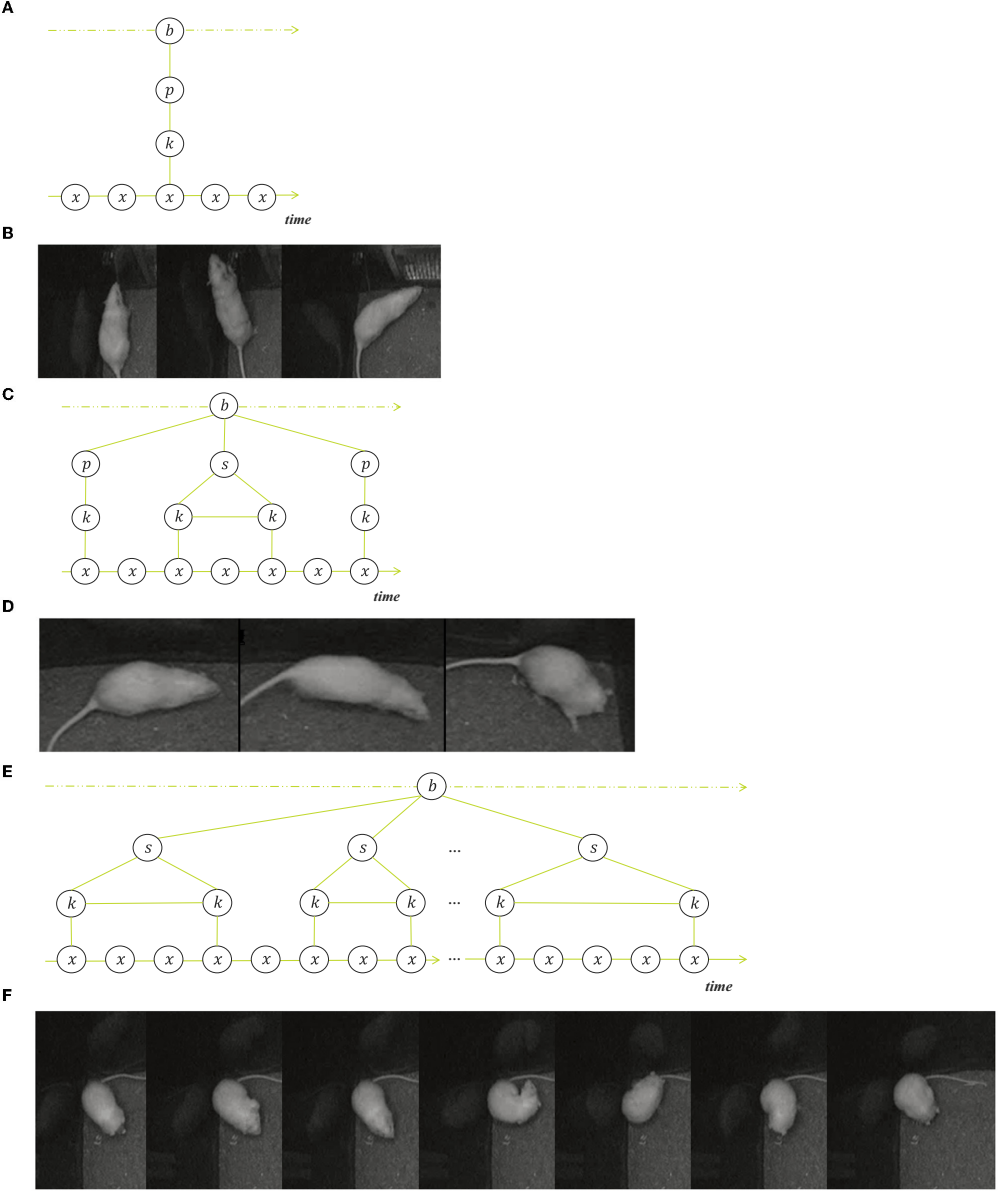
Three examples of structured behavior, each with a schematic representation and a selection of frames from a single event. **(A, B)** Show a point event (rearing), **(C, D)** show an ordered composition (jumping) that consists of three sub events, namely a point event (take-off), a state event (stretched pose) and another point event (landing). **(E, F)** illustrate an unordered sequence of state events (grooming).

We illustrate the composition of behavior into a sequence of transitions, state events and point events with three examples of rodent behavior in Figure 2.

Figures 2A, B show a typical rearing event, where the behavior consists of a transition from the non-rearing key pose before the rearing, toward the short peeking pose in an upright body position, followed by the second transition toward the next non-rearing key pose. What happens often is the detection of a false-positive rearing event when the actual rearing pose does not occur but the animal is shortly retreating to change direction. However, the system detects the transitional movements, i.e., a forward movement or turn followed by a backward movement or turn. The point event in the middle that is defining the event as rearing is missing but the transition samples match most of the samples of the rearing events in the training set. Note that rearing events can also be state events, when the upright position is held for some time.

The next example in Figures 2C, D is a jumping event that starts with the point event of the take-off, followed by a fast-forward movement and the landing as a second point event. These two point events, the take-off and the landing, are essential for the jumping behavior and distinguishes it from mere walking behavior. Yet the majority of the samples in the jumping event are in the fast-forward movement, so the behavior distributions of walking and jumping overlap considerably when all samples are weighted equally.

The third example in Figures 2E, F is a grooming event that is composed of multiple state events that are not strictly ordered although the common sequence is grooming snout, head, fur, genitals. The grooming-snout substate samples overlap considerably with substates of behaviors eating, sniffing and resting, but can nevertheless be identified as grooming because they are surrounded or followed by more outstanding grooming subevents. In this case it is the context of the surrounding substates that determine the decision when made by a human annotator.

#### 3.1.3. Distribution characteristics of rodent behaviors

**Figure 3:**
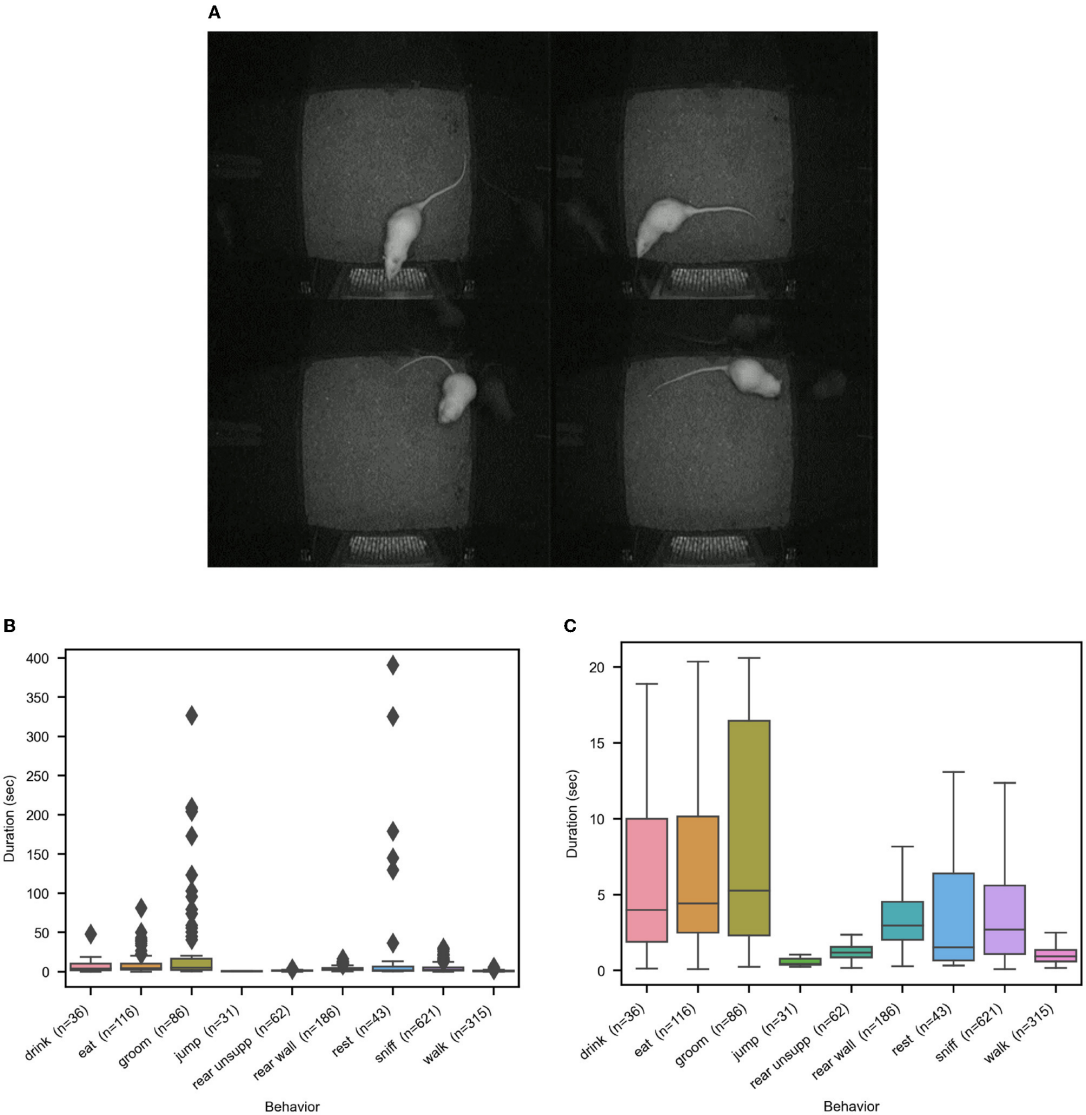
Illustrations of the distribution characteristics of rat behavior that make automated recognition challenging. **(A)** shows four examples of pose confusions. Clockwise, starting upper left, are the confusions (manual label/automated label) sniff/eat, sniff/drink, eat/groom, groom/eat. **(B, C)** show the distributions of the behavior event durations on the entire rat dataset, with and without outliers, to illustrate the big differences across and within behavior classes.

Apart from the challenging demands posed by the composite behaviors, additional characteristics of rodent behavior make automated recognition difficult. These are: high overlap between poses of different behavior classes, high variance between events of the same class, mixture of pose distributions for a subset of classes, unbalance of event frequency distributions hence little training data for rare but important classes, and finally, high variance in event duration, which makes it difficult to set global temporal scales for processing. We give examples of pose overlaps and present behavior event distributions in Figure 3.

### 3.2. Data

To analyze the extent to which automated behavior recognition models are able to model rodent behavior in general and composite rodent behavior in particular, we experiment with two types of data: real rat behavior data and artificial abstracted behavior inspired by real rat behavior.

#### 3.2.1. Rat behavior dataset

The rat behavior dataset was reused from previous work and is described in (Dam et al., 2013). It consists of 25.3 video hours of six Sprague-Dawley rats, each in a PhenoTyper 4500 cage^1^ at 720 × 576 pixel resolution, 25 frames per second and with infrared lighting, hence gray-scale. Subsets of these recordings (~2.7 h in 14 subvideos) were annotated by a trained observer using The Observer XT 10.0 annotation software,^2^ and manually checked and aligned afterwards to ensure frame accurate and consistent labeling. In this study we focused on the nine most frequent behavior classes “drink,” “eat,” “groom,” “jump,” “rest,” “rear unsupported,” “rear wall,” “sniff,” and “walk”. To focus on the dynamics, we applied the same input preprocessing as was used in VAME by Luxem et al. (2022), namely we tracked six body-points using DeepLabCut (Mathis et al., 2018), and aligned and normalized these.

#### 3.2.2. Artificial time-series

In order to experiment with different types of behavior dynamics without suffering from incomplete or incorrect features or insufficient amount of data, we generated artificial time-series of randomly sampled behavioral events, with predefined behavior components and substate dependencies inspired by the rodent behavior components. The sample features, or poses, are drawn from predefined distributions, with configurable variation across and inside events. Components are either point events or states with durations sampled from a distribution, and are concatenated by transition periods of two to eight samples. Per behavior event, we added fluctuations with configurable smoothness, amount and periodicity. As a last step, we added observation noise. The result is a configurable amount of time-series data that we can train the recognition models on, with configurable difficulty, depending on the number of behaviors, number of features, overlap in feature distributions, complexity of behavior structure, and amount of overlap between the constituents of different behaviors. With this procedure we generated two different datasets to experiment with: (1) artificial state behaviors and (2) artificial composite behaviors. The code to construct these datasets is publicly available.^3^ In the code repository, we included the definitions for the artificial datasets used here, as well as an example with four features.

##### 3.2.2.1. Artificial state behaviors

**Figure 4:**
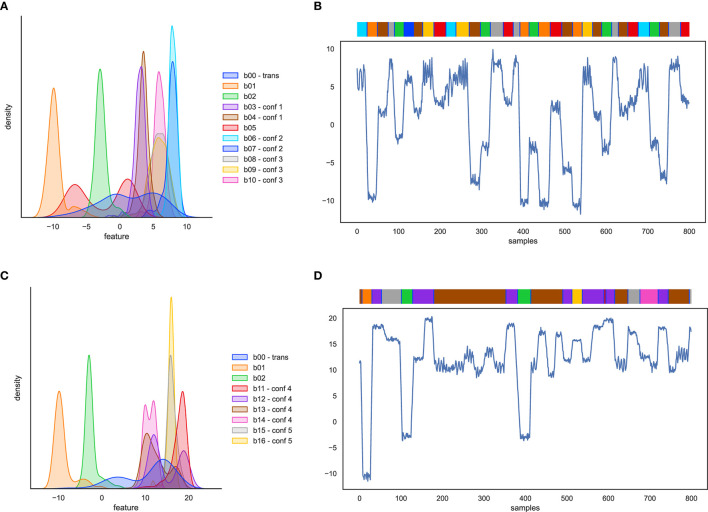
Artificial behavior feature distributions and 1-dimensional time-series example with the behavior event bar on top. **(A, B)** for artificial state behaviors, and **(C, D)** for artificial composite behaviors.

The first artificial dataset contains only state behaviors, modeled after the varying distribution characteristics mentioned in Section 3.1.3. The feature distributions and an example time-series of state behaviors are plotted in Figures 4A, B. The following behaviors are included. First, behaviors with well separated pose (b01, b02), which should be easy to recognize and are added as sanity check. Second, behaviors with poses that are alike (b03, b04; confusion group 1). In real rat data there are behavior pairs have overlapping poses, for instance “drink” and “sniff”. Third, behaviors whose pose distributions are a mixture of poses (b05), for instance as “groom” and “eat”. Fourth, behaviors with uncommon event duration distributions, either long or short (b06, b07; confusion group 2). Examples in rat behavior are “sleep” and “twitch”. Fifth, periodic behaviors (b08, b09 overlapping with behavior b10; confusion group 3). Finally, we inserted pose transition samples between behavior events (b00).

##### 3.2.2.2. Artificial composite behaviors

The second artificial dataset contains two behaviors with well-separated pose (b01 and b02) and additionally the following composite behaviors. First, point behaviors, i.e., defined by key poses of zero or minimal duration, with transitions dependent on the key poses of surrounding events. Point behaviors are hard to detect because they may overlap with samples from state behaviors or with transition samples. An example in the rat behavior data are rearing events, where the surrounding frames are similar to sniffing poses. In the artificial dataset, the point behavior is b11, overlapping with b12. Second, ambiguous subbehaviors in unordered sequences: behaviors defined as an unordered sequence of subbehaviors that have their own distributions, and where some of these subbehavior distributions overlap with other behaviors (behavior1 = *n* x {A or B or X}, behavior2 = {P or Q or X}). In the rat behavior data this corresponds with the overlap between grooming-snout and eating events (b13, overlapping with b14: confusion group 4). Third, ambiguous subbehaviors in ordered sequences: behavior defined by a specific, fixed sequence of subbehaviors, where some of the subbehaviors also occur in the sequence of other behaviors (composite behavior A-X-B vs. behavior P-X-Q). An example in the rat behavior data is jumping behavior that consists of take-off - stretched pose - landing. The stretched pose is also part of a walking sequence (b15, overlapping with b16: confusion group 5). Feature distributions and an example time-series of composite behavior are plotted in Figures 4C, D.

### 3.3. Classification models

We will now describe the two models we used to evaluate the current performance of automated rodent behavior recognition. The first model is a recurrent variational auto-encoder (RNN-VAE) that we applied to all the data. The second is a Transformer model for time-series that we applied to the artificial data.

A good approach is to train a recurrent variational auto-encoder (RNN-VAE) to get a behavior embedding for every short time window of length *T* (*T* = 0.5 s) and use this embedding as input for a small linear network that aims to find *n* behavioral motifs (*n* = 30) from the data. The mapping to the motifs is then used to classify the final behaviors per sample in a supervised manner, using a linear classifier. We followed the network implementation of VAME (Luxem et al., 2022) with an encoder consisting of two bidirectional GRU layers (hidden dimension *h* = 64) and a decoder of one GRU layer (*h* = 32) plus a linear layer to map the input resolution of *T*×*F*, where *F* denotes the feature dimensionality. The embedding size varies with the size of the features: For the rat data (14 features) we used embedding dimension *d* = 30, and for the artificial data with only one feature we use *d* = 6. The output of the encoder is the concatenation of the hidden RNN states. Before passing the output of the encoder to the decoder, a joint distribution is learned and sampled from during training, to ensure better robustness of the embedding. The *n* motifs are learned by including in the loss the clustering-based spectral regularization term [see Luxem et al., 2022 (supporting information), Ma et al., 2019]. In our experiments, we did not train the motifs and behavior classification separately, but instead added a supervised classification head. This means we allowed the network to optimize embedding and motifs for both the decoding and the behavior classification task, by optimizing three losses: a self-supervised reconstruction loss, a clustering loss and a supervised classification loss. During training, the importance of the classification loss is gradually increased.

Note that for supervised classification we could have omitted the motif cluster mapping. We kept it in because we want to investigate the model's ability to learn motifs for the difficult (rare, subtle, composite) behaviors.

As an alternative model, we replaced the RNN-VAE network with a Transformer network derived from LIMU-Bert (Xu et al., 2021), a Bert model for time-series, and applied it to the artificial datasets. The model has four encoder layers, each with four attention heads and a feed-forward layer with hidden size *h*= 80. A linear decoder projects the encoded input back to the original input size *T*×*F*. As in LIMU-Bert, to train the encoder, the input sequence of 20 samples is masked with a contiguous span of samples instead of individual samples to avoid trivial solutions (mask ratio = 0.45), and only the spans are represented and predicted. After reconstruction, the entire original input sequence is encoded without masking and a slice of five samples is classified with a bidirectional GRU classification head (*h* = 30). As before, the reconstruction loss and a supervised classification loss are trained simultaneously.

For all experiments, we performed a hyperparameter search with Optuna (Akiba et al., 2019) to ensure the best possible results. The tuned parameters are learning rate, number of hidden dimensions and the size of the embedding. For the Transformer network we also tuned the mask ratio and the window size of the slice that is sent to classification.

## 4. Results

### 4.1. Modeling rat behavior as switching states

**Figure 5:**
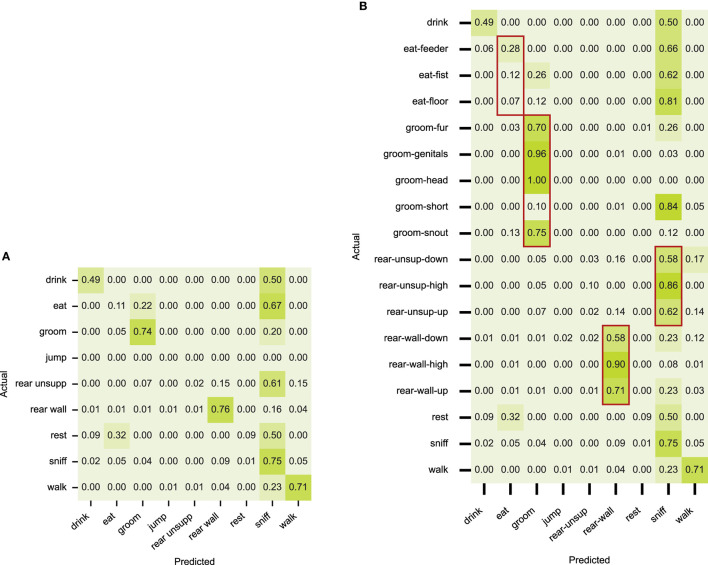
Confusion matrices with classification results on the rat dataset using the supervised RNN-VAE, per behavior on **(A)** and per sub-behavior on **(B)**. The overlaps differ per subbehavior.

The confusion matrices in Figure 5 presents the result of the RNN-VAE model on the rat behavior dataset, calculated from the sequences of the aligned six body-point coordinates per frame. Figure 5A shows the confusions at event level, Figure 5B shows confusions at sub-event level. It is clear that the recognition works well for some of the state behaviors and is less successful for other behaviors. Half of the drinking frames are detected as sniffing, and most of the eating samples are seen as sniffing or grooming. Eating is executed in three different ways: at the feeder, in which case it overlaps with sniffing, or away from the feeder in a sitting pose or off the floor, in a way that is also overlaps with the grooming-snout pose. Nearly all behaviors are confused with sniffing, which is due to overlap in both pose and movement intensity of the very wide distribution of sniffing poses. For a human annotator, it is the context of more explicit behavior that determines the decision. The confusion in resting behavior is because the sequences in the test data are very short compared to the few very long resting periods in the training data, and in different poses. In the detailed results of the rearings, the middle part of the rearing (“high”) is confused differently than the upward and downward movements, which can be due to our observation that rearing events contain a relatively large amount of transitional samples.

Overall we identify four types of confusion. First, the features can be sub-optimal, i.e., incomplete, insufficient or just noisy and incorrect. Next, point behaviors may not be detected. Furthermore, confusion is likely when the relevant context is not picked up. Finally, not all confusions are errors. Transitional samples between states get labeled but are in fact ambiguous ground truth.

### 4.2. Modeling artificial behaviors

**Figure 6:**
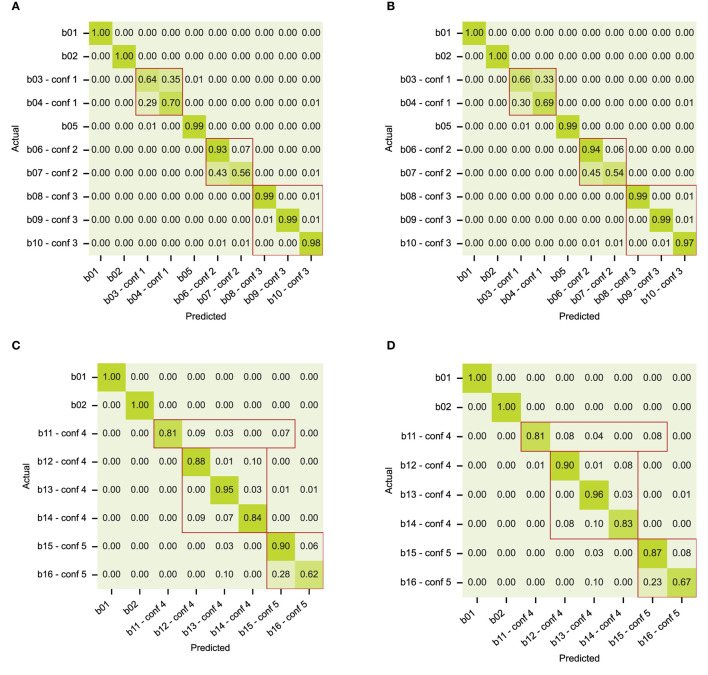
Confusion matrices with classification results on the artificial datasets for **(A)** state behaviors and model RNN-VAE, **(B)** state behaviors and model Transformer, **(C)** composite behaviors and model RNN-VAE, **(D)** composite behaviors and model Transformer. Confusion groups are outlined in red.

The first set of artificial data contains only state behaviors, without structure. Both models can recognize the behaviors equally well, as shown in confusion matrices in Figures 6A, B. The confusion that we see is grouped according to the behavior definitions of the dataset. As expected, classes b01, b02, and b05 are well-separated. The models have difficulty with two of the three confusion groups: confusion group 1 with poses that are alike (classes b03 and b04), and confusion group 2 with uncommon event distributions (classes b06 for long events and b07 for short events). Confusion group 3 with class-specific periodicity (classes b08, b09, and b10) is handled correctly. We conclude that both models can learn state behaviors that have no specific dynamical structure, except for behaviors with class-specific event durations (confusion group 2).

The results on the artificial dataset with composite behavior are presented in Figures 6C, D. This artificial dataset was inspired by the analysis of confusions made in classifying the rat dataset, and contains state behaviors, point behaviors and transitions, as well as state sequences with ambiguous subbehaviors. The behavior definitions overlap in the same way that the rat behaviors do, see the definitions in Section 3.2.2. In the confusion matrix, we see the confusions that we expect, even with a big enough dataset. Again, classes b01 and b02 are well separated. In both models, point behavior b11 (equivalent to “rear”) is confused with state behavior b12 (“sniff”), but also with b15 (“jump”), which is most likely due to the overlap with the transitional poses that comprise most of the b11 context samples. In confusion group 4, behavior b13 (“groom”) was defined as an unordered sequence of substates corresponding to different grooming poses, one of which is overlapping with state behaviors b12 (“sniff”) and b14 (“eat”). See Supplementary Figure 1 for the sub-event level confusion matrix. The models did not use the surrounding context of substates to infer behavior b13. Neither could the models solve confusion in confusion group 5, namely find the conditional context of behavior b15 (“jump”) that separates it from b16 behavior (“walk”).

## 5. Discussion

Currently available automated systems for the recognition of animal behavior from video suffer from lack of robustness with respect to animal treatment and environment setup. In order to be useful in behavioral research, systems must recognize the behaviors of control and treated animals regardless of compound effects on appearance, behavior execution and behavior sequence. Careful analysis of miss-detections in rat behavior recognition lead us to distinguish behaviors into four types of behavior constituents, namely state events, point events and pose transitions, and sequences thereof. To study the performance of recognition models on the different types of dynamics, we created artificial time-series and present results for the most advanced recognition systems.

The classification results on the artificial dataset show that, even with sufficient amount of data with absent noise and ideal annotation quality, and with supervised classification and hyperparameter tuning, the networks are not capable of classifying the composite rodent behaviors, or behavior-specific event durations. Therefore, the solution toward more robust rodent behavior classification is not only to train on more data or to avoid input noise. We also need to improve on how to break down the composition. If models can learn to compress time-series into segments that correspond to behavior constituents, they can analyse segment properties and sequences regardless of the temporal scale of the segments. The usual way of segmenting data into equidistant samples and segments of equal duration is therefore not the best way to segment behavior, and adding the attention mechanism of the Transformer is not enough to overcome this.

Although rodents can switch goal poses instantaneously, they can only change their actual pose in a continuous manner. This makes certain samples more informative then others. Pose changes while changing from one behavior to another are not informative for the behaviors themselves. This is generally true for recordings of intentional agents. How to infer the agent's goal poses is unsolved so far, but if we can discard the uninformative transitional samples we can reduce confusion. One possible way to achieve this is to predict future poses, and take as start and stop pose of the transition the frames that are difficult to predict. Although this seems a good approach, it is very difficult to steer the predictions from the data itself given the amount of variation and valid, possible projections.

With the data compressed into behavior segments and transitions, we would be able to normalize and augment the behavior execution and the behavior sequence which would make classifiers more robust. Breaking down composite behaviors will furthermore increase the detection accuracy of difficult behaviors, for it allows to highlight short yet necessary constituents.

We showed that adding more training data is not sufficient to make progress for several ethologically relevant behaviors, and we argue that understanding the composite nature of animal behavior is necessary to move the field forward. We believe that discarding uninformative pose transitions will reduce confusions and that detection and evaluation of segment sequences will pick up more relevant context. Future research will focus on this direction.

## Data availability statement

The datasets presented in this article are not readily available because the rat behavior dataset is proprietary to Noldus Information Technology. The data necessary to reproduce the results in the manuscript are available upon request and after permission from Noldus Information Technology, with restriction to academic use. The artificial data will be uploaded to the publically accessible data repository of Radboud University. Requests to access the datasets should be directed to info@noldus.nl.

## Author contributions

EV and MV contributed to conception and design of the study. EV conducted the experiments and wrote the manuscript. MV and LN supervised the research. All authors contributed to manuscript revision, read, and approved the submitted version.

## Footnotes

^1^ http://www.noldus.com/phenotyper

^2^ http://www.noldus.com/observer

^3^https://github.com/ElsbethvanDam/artificial_behavior_data (preliminary repository location).

## Supplementary material

Supplementary Figure 1 Sub-behavior level confusion matrices with classification results of model RNN-VAE on the artificial datasets for **(A)** state behaviors and **(B)** composite behaviors. For the composite behaviors, only some of the sub-behaviors overlap with other behaviors. Confusion groups are outlined in red.
