# Supplementary figures and images for: Disentangling rodent behaviors to improve automated behavior recognition
Source: Front Neurosci. 2023 Jul 11;17:1198209. doi: 10.3389/fnins.2023.1198209 (PMC10366600; doi:10.3389/fnins.2023.1198209)

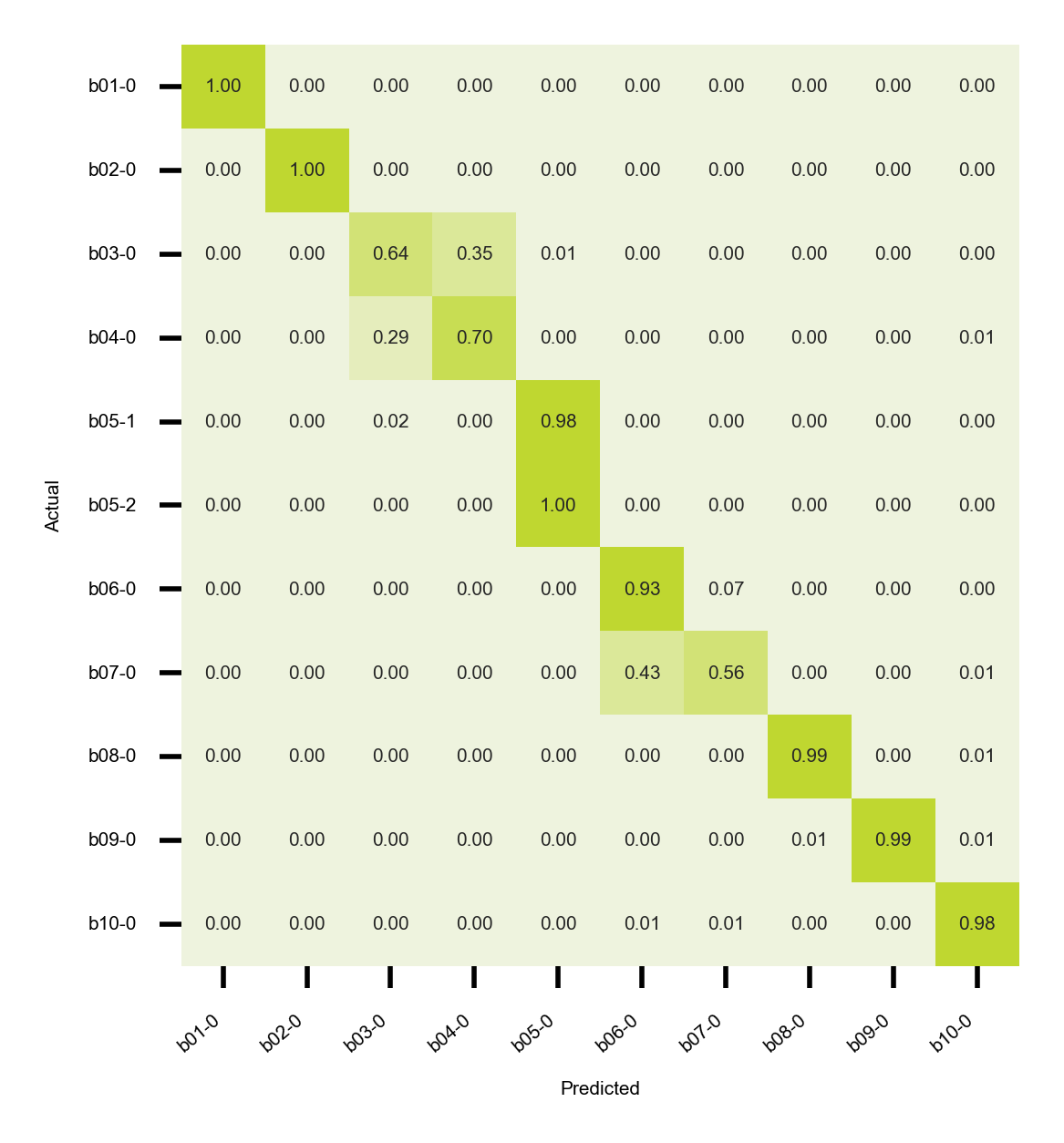

Supplement: Supplementary Figure 1 — Sub-behavior level confusion matrices with classification results of model RNN-VAE on the artificial datasets for (A) state behaviors and (B) composite behaviors. For the composite behaviors, only some of the sub-behaviors overlap with other behaviors. Confusion groups are outlined in red. [file Data_Sheet_1.zip › Supplementary Material Presentation/Suppl_Fig1a_Results_Artif_nostruct_RNNVAE_withgroups_sublevel.tiff]

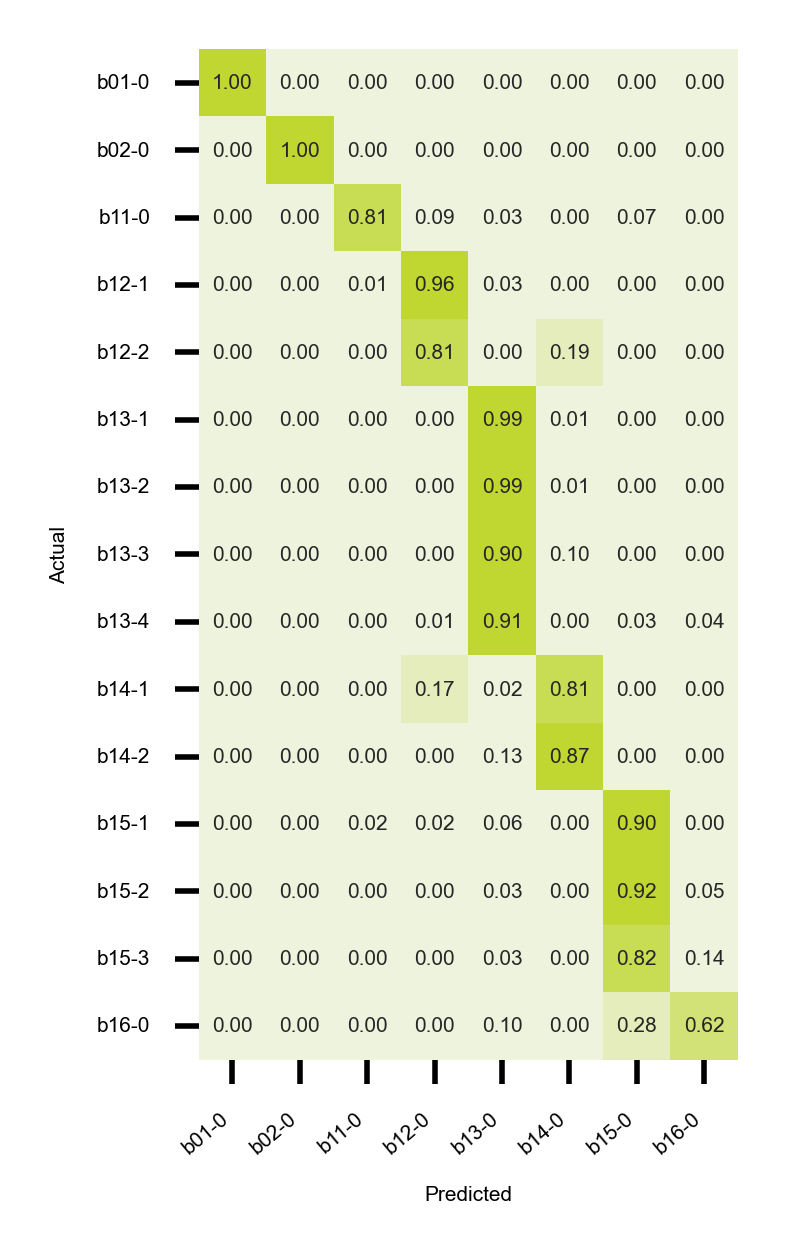

Supplement: Supplementary Figure 1 — Sub-behavior level confusion matrices with classification results of model RNN-VAE on the artificial datasets for (A) state behaviors and (B) composite behaviors. For the composite behaviors, only some of the sub-behaviors overlap with other behaviors. Confusion groups are outlined in red. [file Data_Sheet_1.zip › Supplementary Material Presentation/Suppl_Fig1b_Results_Artif_struct_RNNVAE_withgroups_sublevel.tiff]
